# Layered Double Hydroxide-Based Nanomaterials-From Fundamentals to Applications

**DOI:** 10.3390/nano9081174

**Published:** 2019-08-16

**Authors:** Istvan Szilagyi

**Affiliations:** MTA-SZTE Lendület Biocolloids Research Group, Interdisciplinary Excellence Center, Department of Physical Chemistry and Materials Science, University of Szeged, H-6720 Szeged, Hungary; szistvan@chem.u-szeged.hu; Tel.: +36-62-34-3255

Layered double hydroxides (LDHs) and their composites with various substances represent an important class of materials suitable for several existing and future applications in biological, chemical, and environmental processes. The current widespread interest in these materials is evidenced by recent reviews [1,2,3,4,5] and high-impact journal articles [6,7,8] published since this Special Issue was launched. The advantageous properties of these materials, like biocompatibility, lamellar structure, and compositional diversity, together with the ease of their synthesis even in larger scales, has attracted the interest of a large number of scientists working in academia and in applied disciplines. Such an increased research activity led to progressively growing number of publications in the recent past, seven of which appear in this Special Issue. They include five original research papers [9,10,11,12,13] and two review articles [14,15] from world-class research groups. The topics of these papers are in line with the original goal of the Special Issue, as they range from the synthesis of novel LDHs and LDH-based composites, through their physico-chemical characterization, to applications as fertilizers, drug delivery vehicles, and catalysts. In the following paragraphs, a brief overview of the articles is provided by the guest editor.

In the first paper released in the Special Issue, Ding et al. reported an immobilization procedure for dextranase enzyme on Mg/Fe-LDH [13]. This system holds great promise as a catalyst for the hydrolysis of specific bonds in dextran, which can be utilized in the sugar industry. The work unambiguously pointed out that dextranase adsorption is feasible on the outer surface of the LDH due to electrostatic and hydrophobic interactions between the protein and the surfaces. However, the experimental conditions such as pH and presence of amino acids affect the extent of adsorption significantly. Therefore, the article provides the optimal experimental conditions for the immobilization process of dextranase on Mg/Fe-LDH. Because of the widespread interest in enzyme immobilization, the results should attract significant attention in the scientific and technological communities.

The discussion continues with the contribution of Zhao et al. [11], which is concerned with the development of LDH-based catalytic systems for the reduction of nitrogen oxides. Given the fact that these gases are largely emitted from the industrial combustion of fossils, their capture or catalytic conversion to non-harmful substances is crucial to prevent large-scale environmental pollution. The authors reported the synthesis of an Mn/Al layered double oxide from an Mn/Al-LDH precursor, which was able to act as a catalyst without further modification of its structure by other compounds of catalytic activity. The obtained mixed oxide showed excellent performance, and high yields were achieved even at moderate temperature.

In energy-related research, supercapacitors represent an important class of devices owing to their large power density and long lifespan. The review by Yan et al. [15] summarizes the recent progresses in the design and application of novel Ni/Mn-LDHs as electrode materials for supercapacitors. These types of LDHs are especially promising due to their low cost and high specific capacity. The review discusses synthetic strategies of Ni/Mn-LDHs used for such purposes in the recent past, with special emphasis on LDH-derived films and composites. The development of such supercapacitor electrodes will solve existing problems in energy storage.

Colloid chemistry studies, i.e., comprehensive investigations on charging and aggregation processes in LDH dispersions, were rarely reported in the past, although this issue is critical in all applications taking place in liquid medium. In heterogeneous catalysis by LDHs, for instance, homogeneously distributed primary catalyst particles are required during the run, while they can be removed by aggregation and subsequent sedimentation once the reaction is terminated. The article by Somosi and his co-workers [12] addressed this important issue. In this study, the colloidal stability of Mg/Al-LDH dispersions was tuned by the addition of polyelectrolytes. The study sheds light on the importance of polyelectrolyte adsorption processes on the outer surface of particles and the subsequent changes in the surface charges, which govern the strength of the major interparticle forces. The main novelty of the results is that the formation of polyelectrolyte bilayers using two types of oppositely charged polymers remarkably improved the stability of the LDH dispersions. Due to the high colloidal stability and advanced surface properties necessary for the immobilization of guest molecules, the developed LDH–polyelectrolyte composite is a promising candidate for delivering biomolecules in living systems.

In the next article published in the Special Issue, Qu et al. [14] reviewed the progress made in the field of mechanochemically prepared LDHs. Although liquid phase approaches for LDH synthesis are being used more frequently, LDH preparation by mechanochemical methods often results in the formation of LDH materials of advanced properties, which are useful in adsorption and catalytic processes. These features include high surface chemical activity and abundant surface defects. Concerning these issues, the growing utilization of this synthetic method can be predicted in the future. The authors also discuss that certain mechanochemical processes lead to the formation of agglomerated LDHs, while primary particles of nanoscale size distribution can be obtained by using additives during the top-down synthetic procedures. This review is an excellent collection of the scientific information released recently in the relevant literature and it may provide a stimulus for future projects in the field.

An excellent contribution dealing with the design of surface-modified Mg/Al-LDH particles and their use for nucleic acid delivery was received from Wu and his co-workers [9]. To improve the colloidal stability of the system, the LDH’s outer surface was coated with bovine serum albumin, a strongly adsorbing natural polyelectrolyte. The delivery efficiency was assessed and compared to that of a spherical inorganic particle of different composition. The results unambiguously revealed that both particles are able to quickly deliver the target molecules into cells, and these systems are promising candidates for the development of effective immunotherapy methods for cancer treatment. Such an application of LDH-based materials possesses huge potential in other biomedical treatments too, as has already been indicated by the growing research activity in the field of LDH-based delivery systems.

The last paper included in this Special issue, submitted by Borges et al. [10], is concerned with a different, but also biorelevant topic, namely, the synthesis and characterization of sustainable fertilizers based on Mg/Al- and Mg/Fe-LDH materials. The importance of this topic comes from the fact that new products or methods should be developed to improve nutrient management, which becomes especially important for environmental resilience. A mechanochemical method was used to prepare LDHs doped with dipotassium hydrogen phosphate, and the structure of the obtained composites was studied in detail with well-chosen methods. This approach led to the development of excellent slow-release compounds for potassium (K) and phosphate (P). The release properties of P were further improved by adding carboxymethylcellulose during the mechanochemical synthesis. The results give useful indications for the further design of P/K fertilizers with advanced releasing features, to be used in the agriculture sector.

In conclusion, the contributions of this Special Issue of *Nanomaterials* entitled ‘Layered Double Hydroxide-Based Nanomaterials—From Fundamentals to Applications’ cover a wide range of LDH-related topics. One could learn about novel synthetic methods, strategies for surface modifications, immobilization of biomolecules, catalytic performance of LDH-based composites, and their energy-related applications as electrode materials. It is certain that the goal of the Special Issue has been achieved, since the recent progress in a wide range of LDH-related topics was covered by the articles, which were received from excellent research groups with activity in the field of LDH chemistry. The guest editor believes that by reading these contributions, inspiration for future research will be given to LDH scientists working in any discipline.

## References

[1] Bukhtiyarova M.V. (2019). A review on effect of synthesis conditions on the formation of layered double hydroxides. J. Solid State Chem..

[2] Mishra G., Dash B., Pandey S. (2018). Layered double hydroxides: A brief review from fundamentals to application as evolving biomaterials. Appl. Clay Sci..

[3] Lu P., Liu Y., Zhou T.T., Wang Q., Li Y.S. (2018). Recent advances in layered double hydroxides (LDHs) as two-dimensional membrane materials for gas and liquid separations. J. Membr. Sci..

[4] Forano C., Bruna F., Mousty C., Prevot V. (2018). Interactions between biological cells and layered double hydroxides: Towards functional materials. Chem. Rec..

[5] Wang J.Y., Zhang T.P., Li M., Yang Y., Lu P., Ning P., Wang Q. (2018). Arsenic removal from water/wastewater using layered double hydroxide derived adsorbents, a critical review. RSC Adv..

[6] Taviot-Gueho C., Prevot V., Forano C., Renaudin G., Mousty C., Leroux F. (2018). Tailoring hybrid layered double hydroxides for the development of innovative applications. Adv. Funct. Mater..

[7] Jose N.A., Zeng H.C., Lapkin A.A. (2018). Hydrodynamic assembly of two-dimensional layered double hydroxide nanostructures. Nat. Commun..

[8] Pavlovic M., Nafradi M., Rouster P., Murath S., Szilagyi I. (2019). Highly stable enzyme-mimicking nanocomposite of antioxidant activity. J. Colloid Interface Sci..

[9] Wu Y.H., Gu W.Y., Li L., Chen C., Xu Z.P. (2019). Enhancing Pd-1 gene silence in T lymphocytes by comparing the delivery performance of two inorganic nanoparticle platforms. Nanomaterials.

[10] Borges R., Wypych F., Petit E., Forano C., Prevot V. (2019). Potential sustainable slow-release fertilizers obtained by mechanochemical activation of MgAl and MgFe layered double hydroxides and K_2_HPO_4_. Nanomaterials.

[11] Zhao D., Wang C., Yu F., Shi Y.L., Cao P., Dan J.M., Chen K., Lv Y., Guo X.H., Dai B. (2018). Enhanced oxygen vacancies in a two-dimensional MnAl-layered double oxide prepared via flash nanoprecipitation offers high selective catalytic reduction of NO_x_ with NH_3_. Nanomaterials.

[12] Somosi Z., Pavlovic M., Palinko I., Szilagyi I. (2018). Effect of polyelectrolyte mono- and bilayer formation on the colloidal stability of layered double hydroxide nanoparticles. Nanomaterials.

[13] Ding Y., Liu L., Fang Y.W., Zhang X., Lyu M.S., Wang S.J. (2018). The adsorption of dextranase onto Mg/Fe-layered double hydroxide: Insight into the immobilization. Nanomaterials.

[14] Qu J., Sha L., Wu C.J., Zhang Q.W. (2019). Applications of mechanochemically prepared layered double hydroxides as adsorbents and catalysts: A mini-review. Nanomaterials.

[15] Yan A.L., Wang X.C., Cheng J.P. (2018). Research progress of NiMn layered double hydroxides for supercapacitors: A review. Nanomaterials.

